# In-Situ Chemical Thinning and Surface Doping of Layered Bi_2_Se_3_

**DOI:** 10.3390/nano12213725

**Published:** 2022-10-23

**Authors:** Yan Kang, Yinlong Tan, Renyan Zhang, Xiangnan Xie, Weihong Hua

**Affiliations:** 1 College of Advanced Interdisciplinary Studies, National University of Defense Technology, Changsha 410073, China; 2 College of Computer Science and Technology, National University of Defense Technology, Changsha 410073, China

**Keywords:** two-dimensional materials, bismuth selenide, in-situ chemical thinning, surface doping

## Abstract

As a promising topological insulator, two-dimensional (2D) bismuth selenide (Bi_2_Se_3_) attracts extensive research interest. Controllable surface doping of layered Bi_2_Se_3_ becomes a crucial issue for the relevant applications. Here, we propose an efficient method for the chemical thinning and surface doping of layered Bi_2_Se_3_, forming Se/Bi_2_Se_3_ heterostructures with tunable thickness ranging from a few nanometers to hundreds of nanometers. The thickness can be regulated by varying the reaction time and large-size few-layer Bi_2_Se_3_ sheets can be obtained. Different from previous liquid-exfoliation methods that require complex reaction process, in-situ and thickness-controllable exfoliation of large-size layered Bi_2_Se_3_ can be realized via the developed method. Additionally, the formation of Se nanomeshes coated on the Bi_2_Se_3_ sheets remarkably enhance the intensity of Raman vibration peaks, indicating that this method can be used for surface-enhanced Raman scattering. The proposed chemical thinning and surface-doping method is expected to be extended to other bulk-layered materials for high-efficient preparation of 2D heterostructures.

## 1. Introduction

After successful exfoliation of bulk graphite into atomic-thick graphene [1], increasing fascinated properties derived from 2D materials have been demonstrated in recent decades [2,3,4], such as the superconductivity of twisted bilayer graphene [5], tunable bandgap of the transition-metal dichalcogenides (TMDs) [6], and anisotropic photoelectric properties of black phosphorus [7]. In most situations, these extraordinary electronic and optical properties can be observed only when the thickness of the van der Waals solids decreases to one or a few layers. Taking the few-layer bismuth chalcogenides (A_2_B_3_, “A” represents Bi and “B” represents Se, Te) as typical examples, they exhibit anisotropic electrical properties, such as having the insulated state along the c-axis but the metallic state along the surface [8,9,10]. Due to superior thermoelectric performance, Bi_2_Se_3_ has become one of the most studied topological materials [11]. The thickness of Bi_2_Se_3_ is closely related to electrical properties, such as the thickness-dependent topological phase transition in Bi_2_Se_3_ crystal [12].

Scalable fabrication of high-quality, large-size 2D materials is becoming more and more important for industrial applications [13]. Many preparation methods have been developed to obtain single or few-layer Bi_2_Se_3_ sheets for exploring their properties [14,15,16]. Bottom-up approaches including chemical vapor deposition [17], vapor-solid techniques [18] and solvothermal synthesis [19] are proposed to fabricate few-layer Bi_2_Se_3_ sheets. Multiple up-bottom methods are also developed to realize the exfoliation of bulk Bi_2_Se_3_ into few-layer sheets [15,20,21,22,23]. Although high-quality few-layer Bi_2_Se_3_ can be obtained by mechanical exfoliation with assistance of adhesive force of the tapes or the tip force of the atomic force microscopy (AFM), they also suffer from low efficiency [15]. Liquid exfoliation methods including lithium intercalation [14], electrochemical exfoliation [20,21], and ionic liquid-induced forces receive enormous interest due to the advantages of high efficiency [22]. However, the complex operation procedure, as well as the difficulty for fabricating large-size Bi_2_Se_3_ sheets on target substrates, limits their applications in integrated optoelectronic devices [24]. Therefore, it is crucial to develop a facile method for in-situ exfoliation of layered Bi_2_Se_3_ sheets on various target substrates.

Novel properties can be introduced into 2D materials by surface doping. The doping of Bi_2_Se_3_ has been extensively studied for the regulation of the electric and optical properties [25,26,27]. For example, Sb-Doped Bi_2_Se_3_ nanoplates grown in solution were reported to be ambipolar topological insulators with high carrier mobility [28]. Ag was doped into Bi_2_Se_3_ by melt-growth method to tune the Fermi level of Bi_2_Se_3_ upward [29]. Controllable surface-doping of Bi_2_Se_3_ has been demonstrated to be a powerful method for modulating their physical properties and extending their applications.

Herein an ion-exchange-driven exfoliation method is proposed for in-situ chemical thinning and surface doping of layered Bi_2_Se_3_ sheets on the target substrates, step by step. The thickness of the layered Bi_2_Se_3_ sheets can be regulated from a few nanometers to hundreds of nanometers by controlling the cycling number of chemical thinning. Notably, large-size few-layer Bi_2_Se_3_ sheet with a lateral size more than 200 μm can be achieved via the developed exfoliation method. Importantly, self-shedding of the Bi_2_Se_3_ sheets from the bulk crystal can be induced by the expansion stress derived from the intercalation and cation exchange of heavy Ru^3+^. By contrast, complex operation process and high-power ultrasonication are needed for the exfoliation of layered Bi_2_Se_3_ via previous liquid-exfoliation methods. The proposed chemical thinning and surface doping method may find applications in the exfoliation and modulation of other bulk-layered materials.

## 2. Materials and Methods

### 2.1. Materials and Chemicals

Bulk Bi_2_Se_3_ (>99.99%) was obtained from Six Carbon Technology (Shenzhen, China) with a size of 5 mm × 5 mm. Ruthenium (III) chloride hydrate was purchased from Innochem (99.0%, Beijing, China). Acetone (≧99.0%) and ethanol (≧95.0%) were purchased from Sinopharm (Shanghai, China).

### 2.2. Chemical Thinning Methods

Bulk-layered Bi_2_Se_3_ were micromechanically exfoliated from a synthetic bulk crystal on the SiO_2_/Si substrate. To improve the contact between the Bi_2_Se_3_ sheets and the substrate, the obtained layered Bi_2_Se_3_ was annealed at 100 °C for 2 h under vacuum. Ruthenium (III) chloride (RuCl_3_) solution (5 mM) can be obtained by mixing 6.5 mg ruthenium (III) chloride hydrate and 5 mL acetone at about 50 °C for 1 h. Then, the Bi_2_Se_3_ sheets on the SiO_2_/Si substrate were placed in the Ruthenium (III) chloride solution for chemical thinning until the thickness reaches the designed value. After that, the sample was removed from the solution and rinsed with acetone and ethanol. The thickness of the Bi_2_Se_3_ sheets can be regulated by controlling the cycling number of chemical thinning.

### 2.3. Characterization

Optical images of the Bi_2_Se_3_ sheets with various thickness were taken by a Nikon (ECLIPSE LV 150N, Tokyo, Japan) camera that was focused by a 50× objective lens (Nikon Tu Plan Fluor, Tokyo, Japan) and imaged by FLY-CU3E630SP. The thickness and surface morphology of the samples were characterized by AFM system (Bruker Innova, USA) under an ambient atmosphere operating in the tapping mode. The thickness quoted below is averaged from an interior area of the sample. Surface morphologies of the samples were characterized by scanning electron microscopy (SEM) (MIRA3 TESCAN, Brno, The Czech Republic). X-ray photoelectron spectroscopy (XPS) analysis was carried out using X-ray photoelectron spectrometer (PHI 5000Versaprobe-III, Japan). The Raman spectroscopy were conducted with an assembled system using an exciting laser wavelength of 532 nm. The laser was focused by a 100× objective lens (LEICA DM 2700M, Wetzlar, Germany) before irradiating the samples. The reflected light of the sample was collected into the spectrometer with 1800 lines (ANDOR SR-500i, Britain) on the order of 2 s with 200 averaged spectra.

## 3. Results and Discussion

### 3.1. In-Situ Chemical Thinning and Surface Doping of Bulk-Layered Bi_2_Se_3_

**Figure 1:**
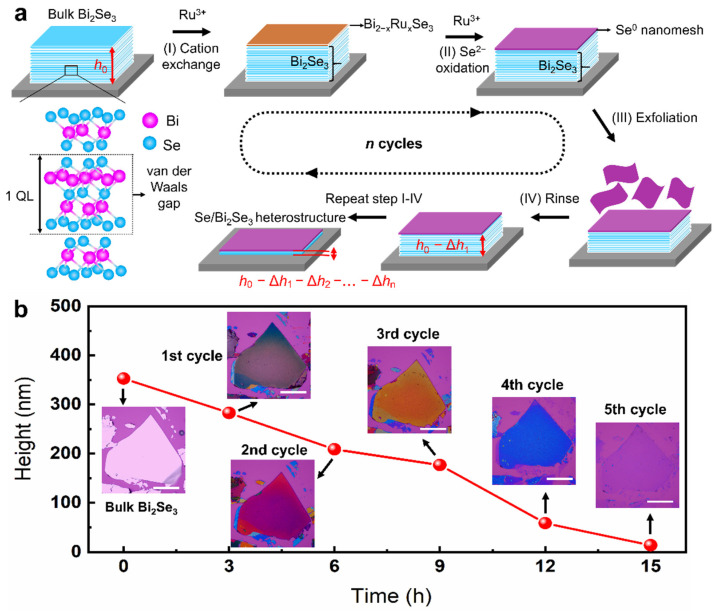
Layer-by-layer chemical thinning and surface doping of layered Bi_2_Se_3_ by varying the reaction cycle: (**a**) Schematics show the chemical thinning process for the fabrication of few-layer Se/Bi_2_Se_3_ heterostructures. (**b**) The thickness of the Se/Bi_2_Se_3_ heterostructures are shown with the variations of the reaction time; in one cycle, the reaction time equals 3 h. The insert images show the color changes of the sample with the variation of reaction time. All scale bars in the inserted images are 50 μm.

Figure 1a depicts the chemical thinning process of bulk Bi_2_Se_3_ into few-layer sheets. First, bulk Bi_2_Se_3_ were transferred onto the target substrates (e.g., SiO_2_/Si) for chemical thinning. Then, the bulk Bi_2_Se_3_ was immersed into the RuCl_3_ solution (5 mM). After that, the solution was heated to 50 °C and stayed for 3 h. At beginning, the Ru^3+^ diffuses onto the surface of Bi_2_Se_3_ and the cation exchange between Ru^3+^ from the solution and Bi^3+^ from the bulk Bi_2_Se_3_ can be induced, forming unstable Bi_2_Ru_2_-xSe_3_ compound. With further increase of the reaction time, Se^2−^ can be oxidized into Se due to strong oxidants, resulting the formation of Se nanomeshes on the surface of bulk Bi_2_Se_3_. During the reaction process, surface wrinkling can be induced by the expansion stress derived from cation-substitution-induced lattice mismatch. The wrinkling layer spontaneously sheds from the bulk Bi_2_Se_3_, leading to a decrease in the sheet thickness.

The thickness of the Se-doped Bi_2_Se_3_ sheets can be regulated by controlling the cycling number of the chemical thinning. Each cycle of the reaction time is fixed at 3 h. As shown in Figure 1b, in-situ chemical thinning of a heart-shaped Bi_2_Se_3_ sheet on a SiO_2_/Si substrate was taken as an example to demonstrate the superiority of the proposed exfoliation method. The thickness of the Bi_2_Se_3_ sheet gradually decreases from 351 to 6 nm by increasing the reaction time from 0 to 15 h (5 cycles). Interestingly, the Se-doped Bi_2_Se_3_ sheets exhibit various colors with the chemical thinning process. The color of pristine Bi_2_Se_3_ sheet changes from off-white to brown after 3 h reaction (1st cycle), and then the color turns to purple-red when the reaction time reaches 6 h (2nd cycle). With further increase of the reaction time to 9 (3rd cycle) and 12 h (4th cycle), the Se-doped Bi_2_Se_3_ sheets exhibit orange and blue color, respectively. Impressively, after 15 h reaction (5th cycle), the color of the Se-doped Bi_2_Se_3_ sheet changes into a purple that is similar to that of the substrate, indicating that the bulk Bi_2_Se_3_ sheet is chemically-thinned into a few layers.

### 3.2. Controllable Exfoliation of Large-Size Layered Bi_2_Se_3_

**Figure 2:**
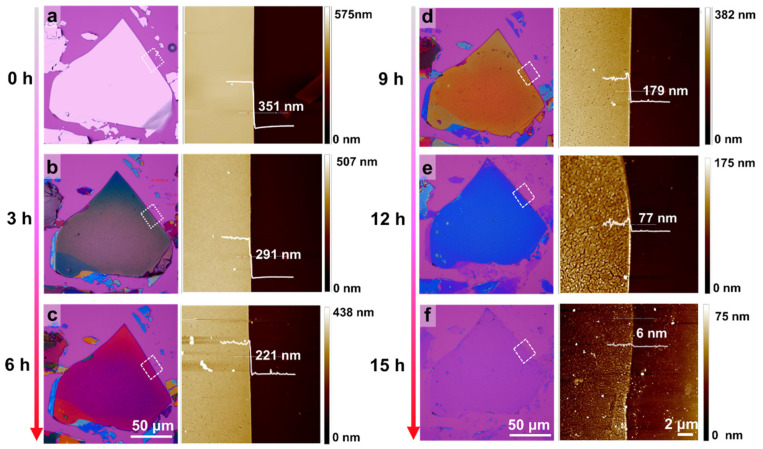
Characterization of the surface morphology and the thickness of the layered Bi_2_Se_3_ sheets with variations of the reaction time. (**a–f**) Optical microscopy images (left) of exfoliated Bi_2_Se_3_ sheets with variations of the reaction time and AFM (right) images of samples in the dotted boxes of the optical microscopy images. (**a**) 0 h. (**b**) 3 h. (**c**) 6 h. (**d**) 9 h. (**e**) 12 h. (**f**) 15 h.

AFM characterization was conducted to see the thickness and morphology evolution of the Bi_2_Se_3_ sheet with increasing the chemical thinning cycles. As shown in Figure 2, the sheet thickness decreases with increases in the reaction time from 3 to 15 h. For example, a decrease of 60 nm in the thickness can be induced by the first cycle treatment (3 h), and the second cycle treatment (6 h) can cause 70 nm chemical thinning. After the fifth cycle treatment (15 h), the thickness can be reduced to ~6 nm. For the heart-shaped Bi_2_Se_3_ sheet with a lateral size of ~150 μm, the average rate of the chemical thinning is about 23 nm/h.

These results demonstrate that the developed chemical thinning method is suitable for in-situ layer-by-layer exfoliation of large-size layered Bi_2_Se_3_, and the thickness of the Bi_2_Se_3_ can be regulated from a few nanometers to hundreds of nanometers via changes in the reaction time. The color evolution of the sheet with increases in the reaction time can also be attributed to the decreasing sheet thickness resulting from the chemical thinning. Notably, the surface of the pristine Bi_2_Se_3_ sheet is smooth and the edge is sharp (Figure 2a), while numerous nanoparticles and nanomeshes can be observed on the surface of the sheet after chemical thinning (Figure 2b–f).

### 3.3. Raman Analysis of the Exfoliated Samples

**Figure 3:**
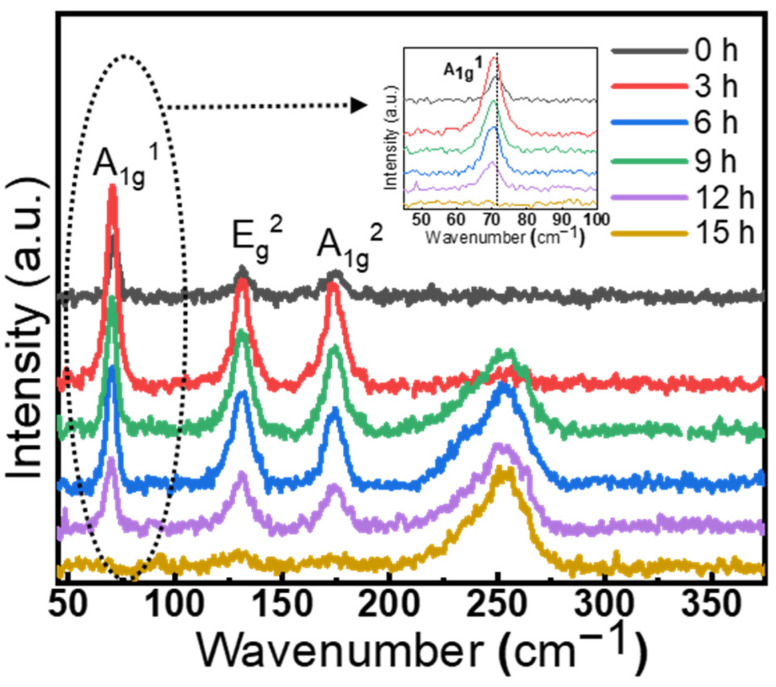
Raman spectra of the samples with variation of the reaction time. Insert: Enlarged view of the A_1_g^1^ peaks.

Raman characterizations of the exfoliated sheets were carried out to investigate the effect of chemical thinning on the structure of the layered Bi_2_Se_3_. Figure 3 shows the Raman spectroscopy of the pristine Bi_2_Se_3_ crystal and the exfoliated sheets after chemical thinning of various time durations. The pristine bulk Bi_2_Se_3_ exhibits three characteristic Raman peaks including 72, 131 and 173 cm^−1^, which can be assigned to the A_1g_^1^ mode (out of plane stretch), E_g_^2^ mode (in-plane stretch) and A_1g_^2^ mode (out of plane stretch) [30,31]. After the first cycle chemical thinning (3 h), the sample also shows typical Raman peaks of Bi_2_Se_3_, but the intensity of characteristic peaks increases remarkably, which can be attributed to the formation of nanoparticles and nanomeshes on the surface. The formation of nanostructures generates numerous nanoscale gaps that are beneficial for surface-enhanced Raman scattering. It is worth noting that a new peak at 253 cm^−1^ appeared after chemical thinning, which can be assigned to the characteristic peak of amorphous Se [32,33,34,35]. This result suggests the formation of amorphous Se on the surface during the chemical thinning process, which will be further demonstrated by the latter XPS analysis. Impressively, the characterized peaks of Bi_2_Se_3_ almost disappeared after the fifth cycle of chemical thinning (15 h), and only the Raman signal of amorphous Se can be observed. This result suggests that ultrathin Se films can be obtained when the reaction time is enough.

With increase of the reaction time to 12 h, the intensities of A_1g_^1^, E_g_^2^ and A_1g_^2^ modes reduce due to the decrease of the sheet thickness, while, within the former 12 h, the intrinsic Bi_2_Se_3_ characteristic peak in the reacted Bi_2_Se_3_ was enhanced relative to the intrinsic Bi_2_Se_3_, presenting surface-enhanced Raman. It is worth mentioning that the peak of A_1g_^1^ shows a ∼3 cm^−1^ shift to lower wavenumber compared to that of pristine Bi_2_Se_3_ (Figure 3 insert). This shift can be attributed to the lower degree of the vibrations in the exfoliated Bi_2_Se_3_, since the A_1g_ modes that correspond to the out-of-plane vibrations of the Se and Bi atoms parallel to the c-axis are very sensitive to the thickness [36,37,38]. Besides, the broadening of the E_g_^2^ peak can be observed for the exfoliated samples, which may be caused by the enhancement of electron-phonon coupling in the few QL regime [30].

### 3.4. Surface Morphology and Element Content Analysis of the Exfoliated Samples

**Figure 4:**
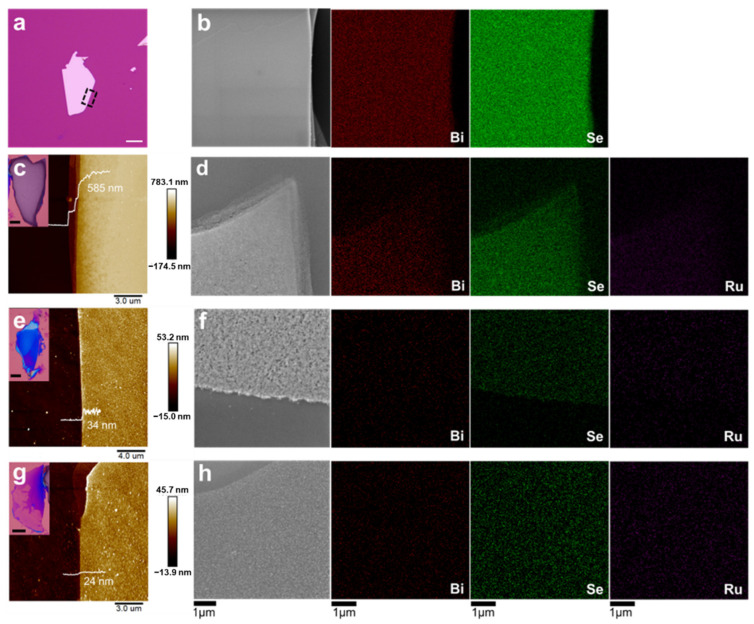
Surface morphology and element analysis of the exfoliated samples: (**a**) optical image of the pristine Bi_2_Se_3_ sheet without chemical thinning; (**b**) SEM image of the pristine Bi_2_Se_3_ sheet and the corresponding EDS mapping for Se and Bi; (**c**) optical and AFM images of an exfoliated sample with a thickness of 585 nm, and (**d**) corresponding SEM image and EDS mapping; (**e**) optical and AFM images of an exfoliated sample with a thickness of 34 nm, and (**f**) corresponding SEM image and EDS mapping; (**g**) optical and AFM images of an exfoliated sample with a thickness of 24 nm, and (**h**) corresponding SEM image and EDS mapping. All of the scale bars in the optical images are 20 μm.

To figure out the element composition of the exfoliated samples, SEM and EDS mapping were carried out to characterize the surface morphology and element contents. The optical and SEM images in Figure 4a,b show that the pristine Bi_2_Se_3_ sheet without chemical thinning has a smooth surface. The EDS mapping result demonstrates that the atomic ratio of Bi to Se is ~2/3, which corresponds to the stoichiometric ratio of Bi_2_Se_3_. After chemical thinning, the formation of nanoparticles and nanomeshes can be induced on the surface of the exfoliated samples, forming rough coatings (Figure 4c–h). Additionally, Ru element can be observed on the surface of the exfoliated samples after chemical thinning. Compared with the exfoliated sample with a thickness of 585 nm, the mapping signals for Bi and Se decreases with reducing the sheet thickness to 34 and 24 nm (Figure 4f–h).

**Figure 5:**
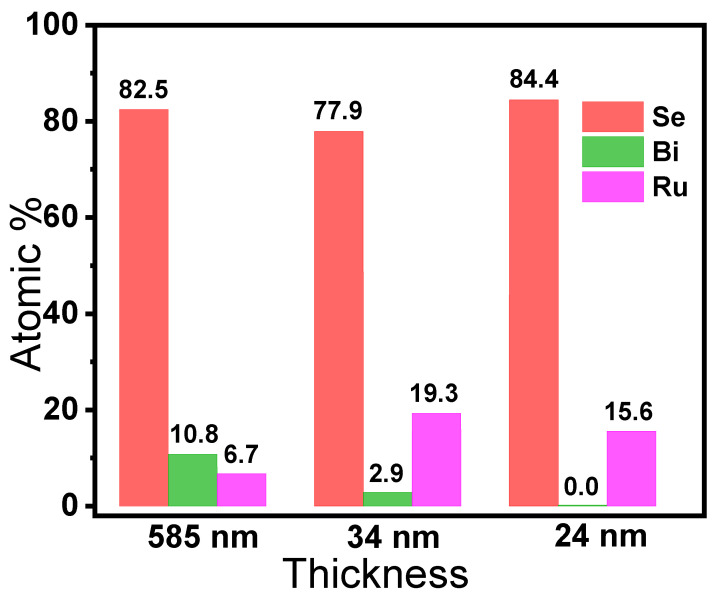
The atomic content of Se, Bi and Ru of the exfoliated samples with associated thicknesses.

As shown in Figure 5, the atomic ratio of Se to Bi is no longer 1.5 for the exfoliated samples. For the exfoliated sample with a thickness of 585 nm, the atomic ratio of Se to Bi is about 7.6. When the sheet thickness decreases to 34 nm, the ratio further increases to 26.9, which is much higher than that of the pristine Bi_2_Se_3_. Impressively, the atomic content of Bi declined, even to 0%, when the sheet thickness decreased to 24 nm. By contrast, the atomic content of Se is always higher than 77%, regardless of the decrease of sheet thickness. These results demonstrate that cation exchange between Bi^3+^ and Ru^3+^ can be induced during the chemical thinning process. The chemical states of various elements will be further verified by XPS characterization in the next section.

### 3.5. XPS Characterization of the Exfoliated Samples

**Figure 6:**
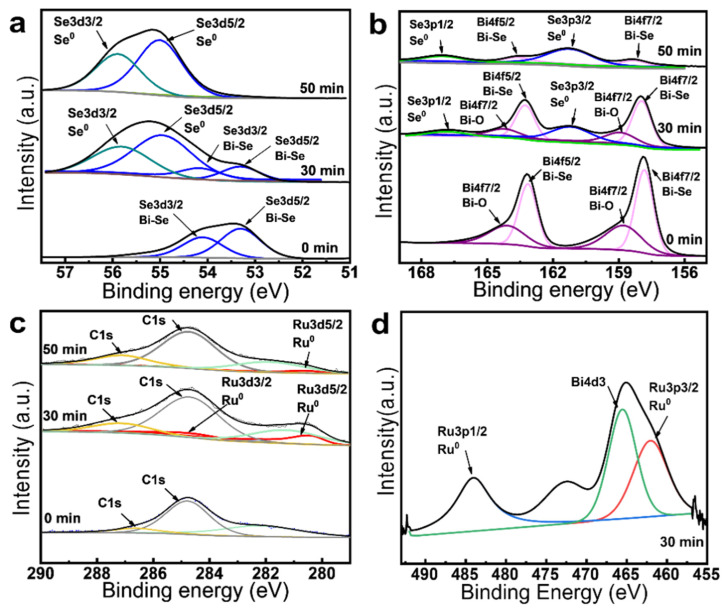
XPS characterization of the pristine and exfoliated samples: (**a**) Se3d; (**b**) Bi4f; (**c**) C1s and Ru3d; these samples were treated with 5 mM RuCl_3_ solution with a duration of 0, 20 and 50 min. (**d**) Ru3p, having been treated with 5 mM RuCl_3_ solution with a duration of 30 min.

The chemical compositions of the exfoliated samples during the chemical thinning were traced by XPS to investigate the surface chemical states. To prepare the exfoliated samples, the pristine bulk Bi_2_Se_3_ sheets were immersed into 5 mM RuCl_3_ solution with durations of 30 and 50 min, respectively. Figure 6 shows the characterized XPS peaks of the pristine Bi_2_Se_3_ and the exfoliated samples. All the peaks are calibrated by the reference carbon peak at 284.8 eV. For the pristine Bi_2_Se_3_ without chemical thinning (0 min), two characterized peaks at 157.8 and 163.1 eV that represent Bi4f7/2 and Bi4f5/2 are observed (Figure 6b). Besides, two peaks at 53.3 and 54.1 eV appear, which can be assigned to Se3d5/2 and 3d3/2, demonstrating the chemical states of layered Bi_2_Se_3_ [33,34].

After immersing the bulk Bi_2_Se_3_ into the RuCl_3_ solution, the intensity of Se3d5/2 and 3d3/2 peaks decreased rapidly with the reaction time while the peaks of Se^0^ clearly increased (Figure 6a). The increase of Se^0^ with the soaking time could be further demonstrated from the increase of Se3p3/2 (161.2 eV) and Se3p1/2 (166.8 eV) in Figure 6b [39]. These results indicate that Se^2-^ tends to be oxidized to zero-valent Se^0^ during the chemical thinning process. As shown in Figure 6b, the peaks of Bi4f7/2 and 4f5/2 decrease with extensions of the soaking time, indicating the dissociation of Bi^3+^ from Bi_2_Se_3_, which is in agreement with the result of the EDS mapping. The dissociation of Bi^3+^ is resulted by the cation exchange between Bi^3+^ and Ru^3+^ [40,41]. Similarly, the substitution of Bi^3+^ of Bi_2_Se_3_ with Cu^+^ cation has been demonstrated in previous study [38]. When the reaction time reaches more than 30 min, two peaks at 280.5 eV and 284.6 eV that represent the Ru3d5/2 and Ru3d3/2 can be observed, suggesting the formation of zero-valent Ru^0^ (Figure 6c). This result can be further confirmed by the characterized peaks of Ru3p3/2 (462.0 eV) and Ru3p1/2 (484.05 eV), as shown in Figure 6d [42].

## 4. Conclusions

In summary, a facile and efficient chemical thinning method is proposed for layer-by-layer in-situ exfoliation and surface doping of large-size bulk Bi_2_Se_3_. Layered Se-doped Bi_2_Se_3_ sheets with tunable thickness ranging from a few nanometers to hundreds of nanometers can be achieved by controlling the reaction time. As opposed to previous liquid-exfoliation methods that require complex reaction processes, thickness-controllable exfoliation of large-size layered Bi_2_Se_3_ can be realized via the developed method. In addition, the formation of Se nanomeshes on the Bi_2_Se_3_ sheets remarkably enhance the intensity of Raman peaks, demonstrating that the proposed chemical thinning method may find applications in surface-enhanced Raman scattering. The developed method is expected to be extended in a controllable manner to other bulk-layered materials for highly efficient preparations of 2D heterostructures for diverse applications.

## Data Availability

Data sharing not applicable.
